# Hierarchical self-assembly of squaraine and silica nanoparticle functionalized with cationic coordination sites for near infrared detection of ATP

**DOI:** 10.1038/srep43491

**Published:** 2017-02-27

**Authors:** Ruizhi Feng, Weining Shi, Dejia Wang, Jia Wen, Hongjuan Li, Shiguo Sun, Yongqian Xu

**Affiliations:** 1 Shaanxi Key Laboratory of Natural Products & Chemical Biology, College of Chemistry & Pharmacy, Northwest A&F University, Yangling, Shaanxi, 712100, P. R. China

## Abstract

Optical activity of hierarchical supramolecular assemblies based on organic dyes would create multiple functional architectures. In this work, three kinds of silica nanoparticles with or without functional groups were synthesized. For the first time, silica nanoparticles can induce positively charged squaraine (SQ) to aggregate to form supramolecular assemblies. Adenosine-5′-triphosphate (ATP) as building blocks was absorbed on the surface of silica nanoparticles through metal-anion coordination and electrostatic interactions, in which the aggregates of **SQ** was transferred to monomer. The thickness being composed of ATP and **SQ** on the outside of nanoparticles is about 5 nm. These supramolecular assemblies showed selective turn-on fluorescence response to ATP in near infrared (NIR) region over other ions through metal-anion coordination and electrostatic interactions. These functional silica nanoparticles possessing many advantages provide proof-of-principle “seed crystals” for construction of supramolecular assemblies and platforms for sensing with facile performance.

The induction of optical activity of supramolecular assemblies based on organic dyes by external stimulus for construction of hierarchical supramolecules and specific sensing is a subject of considerable and growing interest, which represents a powerful and fascinating tool for the creation of multiple functional architectures^1^,^2^,^3^. Squaraine (**SQ**), an interesting class of dyes possessing sharp and intense absorption and fluorescence in the red to near infrared region, that shown to self-assemble into aggregations, are attractive due to their various architectures with rich spectral properties^4^,^5^. In solution, **SQ** dyes are known to be assembled into an ordered structure, with chromophores either in a parallel-oriented fashion (H-aggregate) or in a head-to-tail arrangement (J-aggregates). The J-aggregates give red-shifted absorption bands and enhanced luminescence (as compared to monomer), while H-aggregates exhibit blue shifted absorption bands and poor emission^6^,^7^. Depending on surrounding medium conditions such as solvents and ionic strength, the degree and type of aggregates are different, leading to different optical absorption and emission properties of **SQ**^8^,^9^. Correspondingly, the process of supramolecular assemblies based on tuning of **SQ** aggregates can be tracked through spectra response. Although many methods have been reported to tune and construct **SQ** supramolecular assemblies, most of them take advantage of specific host-guest and electrostatic interactions using macrocyclic molecules cucurbit[n]uril and negatively charged materials^10^,^11^,^12^,^13^. In addition, the further drive of these assembles to produce optical response for target recognition is limited to competitive guest of 1-aminoadamantane^10^,^11^ or macromolecule of protein^12^,^13^, which blocks the extensive use of these supramolecular assemblies (Fig. 1a,b). Therefore, it is of importance to explore new and general approaches to efficiently set up supramolecular assemblies of **SQ**, and be further used for small molecule sensing. Mesoporous silica nanoparticles (MSN) mainly regarded as delivery carries have been widely used in recent years due to the advantages in large surface area, ease of functionalization, chemical stability and the presence of a highly ordered porous network^14^. Although MSN can not only load cargoes but also control the release of entrapped species upon triggering of stimuli, as potential trigger MSN have not yet been reported to construct supramolecular assemblies of dyes. Herein, for the first time we found that MSN with or without functionalized groups can control the aggregates of squaraine to build up supramolecular assemblies. Moreover, the generated assemblies can be applied for detection of small molecule.

Adenosine-5′-triphosphate (ATP), being used as building block^15^, plays important roles in various cellular activities including muscle contraction, transporting process of proteins, modulation of ion channels and activating of cascade signal. As energy currency, ATP is also involved in DNA replication and transcription. Deficiency in the ATP level is associated with disease states such as Parkinson’s disease, angiocardiopathy, ischemia, hypoglycemia and some malignant tumors^16^,^17^. Therefore, as the same importance for reactive oxygen species (ROS) detection described in literatures^18^,^19^,^20^, it is important to develop simple methods to selectively detect ATP for study of biological process and diagnosis of diseases. Although MSN-based fluorescence methods have been used for ATP sensing, these systems in which dyes were doped into or covalently linked with MSN suffer from the leakage of dye and complicated modification of surface^21^,^22^. Furthermore, these assemblies emit short wavelength fluorescence and cannot detect ATP in near infrared (NIR) region. For biological and clinical application, NIR fluorescent sensors are highly desirable because they can effectively avoid photodamage, scattering light and serious interference from short wavelength emission of biological media^23^,^24^,^25^. Thus, developing a simple but effective fluorescent self-assembly based on MSN for NIR detection of ATP remains a challenge. ATP contains a chain with four negative charges arising from the attached phosphate groups, which can interact with molecules with cationic groups. On the other hand, the phosphate groups of ATP can easily coordinate with Zn^2+^ ions, especially DPA-Zn^2+^ (complex of ligand 2,2′-dipicolylamine with Zn^2+^) through metal-anion coordination interaction^26^,^27^,^28^. Herein, silica nanoparticles functionalized with positively charged quaternary ammonium groups (**SiNPs-N**^+^) and DPA-Zn^2+^ recognized sites (**SiNPs-DPA@Zn**^**2**+^) were synthesized for detection of ATP. For comparison, silica nanoparticles without functional groups on their surface (SiNPs) were also synthesized. The abilities of these three kinds of silica nanoparticles as trigger to construct supramolecular assemblies of squaraine were also evaluated. Either SiNPs or positively charged silica nanoparticles can induce positively charged squaraine to aggregate in aqueous solution, which is sharp different from the reported systems dependent on electrostatic interactions (Fig. 1c).

## Results and Discussion

Preparation and characterization of SiNPs, SiNPs-DPA@Zn^2+^ and SiNPs-N^+^ SiNPs-DPA@Zn^2+^ and SiNPs-N^+^ were prepared by reversed-phase microemulsion method^29^. These well-defined and monodispersed functional silica nanoparticles can be clearly observed in the scanning electron microscopy (SEM) images (Fig. S1B–C) and transmission electron microscopy (TEM) images (Fig. S2). According to the SEM and TEM images, the average diameters of SiNPs-DPA@Zn^2+^ and SiNPs-N^+^ were both 60 nm ± 4 nm. Compared with unmodified silica nanoparticles (SiNPs, Fig. S1A), the diameters of functional silica particles show the increasing of about 10 nm^30^. The average solvodynamic diameters of SiNPs, SiNPs-DPA@Zn^2+^ and SiNPs-N^+^ were about 140, 160, 160 nm through the dynamic light scattering (DLS), respectively (Fig. S3). It is reasonable that the hydrodynamic diameters of the particles estimated by DLS were larger than those by SEM and TEM. The comparison of fourier transform infrared (FT-IR) spectra among SiNPs, SiNPs-DPA@Zn^2+^ and SiNPs-N^+^ can further confirm the presence of DPA and quaternary ammonium groups on the surface of silica nanoparticles (Fig. S1D). Newly emerged absorption bands at 1696 cm^−1^ (stretching vibration of the C = N in the pyridine ring of the DPA) and 1487 cm^−1^ (bending vibration of C-H) indicated DPA and quaternary ammonium were successfully introduced on silica nanoparticles. Zeta-potentials of **SiNPs-DPA@Zn**^**2+**^**, SiNPs-N**^**+**^ and **SiNPs** were measured to be 18.6, 15.7 and −32.4 mV, respectively (Fig. S4). The introduction of positively charged DPA-Zn^2+^ or quaternary ammonium groups makes the Zeta-potential of nanoparticles change from negative to positive. X-ray photoelectron spectroscopy (XPS) was another important method for determining the surface composition. The XPS spectra of SiNPs-DPA@Zn^2+^, SiNPs-N^+^ and SiNPs were further analyzed (Fig. S5). The peaks at 1022.0 eV (Zn 2p), 402.0 eV (N 1 s) and 103.0 eV (Si 2p) are attributed to Zn, N and Si, respectively. All these nanoparticles were dispersed in water and no precipitates were observed (Fig. S6A). A color shown by **SiNPs-DPA@Zn**^**2+**^ before and after centrifugation is obviously different from that of the SiNPs, which also proves the presence of DPA-Zn^2+^ (Fig. S6). These results suggest that **SiNPs-DPA@Zn**^**2+**^ and **SiNPs-N**^**+**^ were successfully synthesized.

### The spectra changes of SQ upon treatment with SiNPs, SiNPs-DPA@Zn^2+^ and SiNPs-N^+^ in different solutions

**Figure 1:**
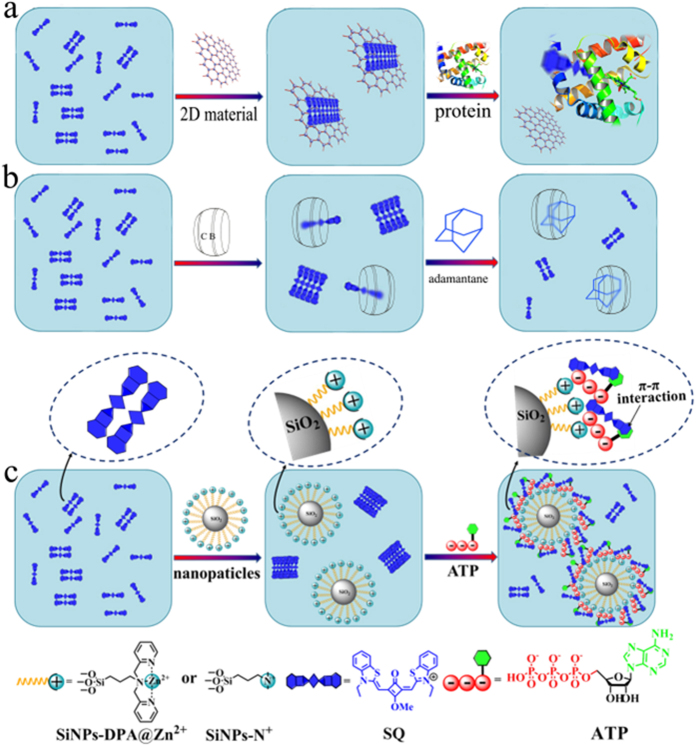
Two known approaches (**a**,**b**) and our new strategy (**c**) of possible assembled assay between functionalized silica nanoparticle (**SiNPs-DPA@Zn**^**2**+^ or **SiNPs-N**^+^), **SQ** and ATP.

**Figure 2:**
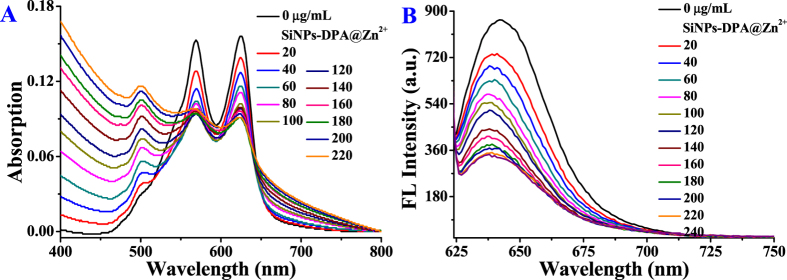
UV-Vis absorption spectra (**A**) and fluorescence spectra (**B**) changes of **SQ** (5 μM) in phosphate buffer (PBS, 10 mM, pH 7.2) upon addition of **SiNPs-DPA@Zn**^**2**+^.

In PBS buffer (10 mM, pH 7.2) solution, **SQ** revealed two main absorption peaks at 625 and 568 nm, which are assigned to monomer and dimer, respectively (Fig. S7)^12^. Upon addition of SiNPs, the absorption intensities of monomer and dimer peaks decreased, and a new absorption peak at 508 nm occurred, which is attributed to H-aggregates of **SQ**^12^. Upon addition of **SiNPs-DPA@Zn**^**2**+^ or **SiNPs-N**^+^, similar absorption spectra changes of **SQ** were observed (Fig. 2 and S8). These results indicated that silica nanoparticles with or without functional groups can induce **SQ** molecules to transform from monomer to aggregates. It should be noted that positively charged **SQ** are easily triggered to aggregates by 2D materials with negatively charged groups^12^,^13^. Seen from Fig. 2A, S7B and S8A, **SiNPs** were found to more easily induce **SQ** to aggregate because there are electrostatic interactions between **SiNPs** and **SQ**. To our surprise, **SiNPs-DPA@Zn**^**2**+^ or **SiNPs-N**^+^ can overcome electrostatic repulsion to induce **SQ** to aggregates. It is presumed that the hydrophobic pores of silica nanoparticles direct hydrophobic **SQ** to aggregate along their surface through hydrophobic and ion-polar interactions^11^. As illustrated in Fig. 1c, silica nanoparticles induce **SQ** to aggregate to form 2D supramolecular assemblies. The fluorescence intensity of **SQ** gradually decreased with addition of silica nanoparticles because of the aggregation-caused quenching (ACQ) property of **SQ**^12^. As the concentration of **SiNPs-DPA@Zn**^**2**+^ reaches to 220 μg/mL, about 2.6-fold decreasing of fluorescence intensity at 637 nm was observed (Fig. 2B).

### The spectra changes of SQ in the presence of SiNPs, SiNPs-DPA@Zn^2+^ and SiNPs-N^+^ in different solutions upon addition of ATP

**Figure 3:**
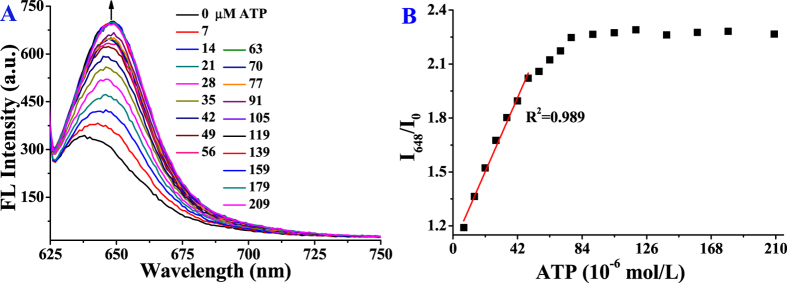
Fluorescence spectra change (**A**) and relative fluorescence intensity change (I_648_/I_0_) (**B**) of **SQ** (5 μM) in the presence of **SiNPs-DPA@Zn**^**2**+^ (220 mg/L) in PBS (10 mM, pH 7.2) upon addition of ATP.

For the assemblies where the aggregation of dye was triggered by electrostatic interactions, only macromolecules such as proteins can cause the dynamic shift of aggregates of dye^12^. For the supramolecular assemblies of **SQ** with silica nanoparticles, small molecule ATP was selected to evaluate the ability in different solutions. Upon addition of ATP, the spectra of **SQ** in PBS buffer solution (10 mM, pH 7.2) or HEPES buffer solution (10 mM, pH 7.2) in the presence of **SiNPs-N**^+^ showed no detectable change (Fig. S9–10). It is possible that electrostatic interactions between ATP and **SQ** and/or **SiNPs-N**^+^ are too weak and easily affected by buffer media^31^. In water instead of buffer solution, the addition of ATP to **SiNPs-N**^+^ **@SQ** caused an increase of the absorption intensities at 625 and 528 nm (Fig. S11). ATP shows the ability of triggering **SQ** in suprmolecular assemblies to convert from aggregates to monomer and dimer. Correspondingly, the fluorescence intensity at 637 nm increased with addition of ATP (Fig. S12). As 120 μM of ATP was added, the fluorescence intensity levelled off, at which the fluorescence intensity of **SQ** recovered up to 28.0% of that of **SQ** alone. The relative fluorescence intensities (I_637_/I_0_) were linearly proportional to the ATP concentrations (10–120 μM, R^2^ = 0.993), and a detection limit (3σ/slope) for ATP was calculated to be about 3.56 μM. The spectra responses of **SQ**-**SiNPs-DPA@Zn**^**2**+^ to ATP in PBS buffer solution (10 mM, pH 7.2), however, are similar to those of **SiNPs-N**^+^ **@SQ** to ATP in water (Fig. 3 and S13). The metal-anion coordination interaction is stronger than electrostatic interaction, enabling ATP to induce the conversion of **SQ** from aggregates to monomer in buffer solution while not be affected by media. The relative fluorescence intensity of **SQ**-**SiNPs-DPA@Zn**^**2**+^ solution at 648 nm (I_648_/I_0_) was linearly proportional to the concentration of ATP over the range from 7 to 49 μM (R^2^ = 0.989, Fig. 3B). The detection limit (3σ/slope) for ATP was estimated to be about 0.87 μM, which is much lower than that of intracellular concentrations (1–10 mM). In addition, the relative fluorescence intensity changes of **SQ**-**SiNPs-DPA@Zn**^**2**+^ solution at 648 nm (I_648_/I_0_) with increasing concentration of ATP under different pH values were investigated. As shown in Fig. S14, the buffer solution with pH value of 7.2 is optimal sensing condition for ATP response no matter from the linear response region or sensitivity. As ATP is added to the solution of supramolecular assemblies, the phosphate anion of ATP is attracted by DPA-Zn^2+^ and quaternary ammonium groups on functional silica nanoparticles (**SiNPs-DPA@Zn**^**2**+^ and **SiNPs-N**^+^) via metal-anion coordination and electrostatic interaction, respectively. The ability of silica nanoparticles to direct **SQ** to aggregate is shielded by the ATP absorbed on surface of silica nanoparticles, resulting in the collapse of supramolecular assemblies. Meanwhile, ATP can interact with **SQ** through electrostatic and π-π interactions^32^. These interactions lead to the dispersion of **SQ**, producing the turn-on fluorescent response to ATP in NIR region. In the presence of ATP, original supramolecular assemblies shift to new hierarchical assemblies.

**Figure 4:**
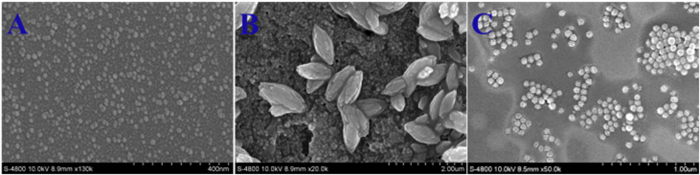
SEM images of **SQ** (**A**) in the presence of **SiNPs-DPA@Zn**^**2**+^ before (**B**) and after (**C**) addition of ATP.

In order to gain insight into the response process, the morphologic changes of supramolecular assemblies (**SQ-SiNPs-DPA@Zn**^**2**+^ and **SQ-SiNPs-N**^+^) before and after addition of ATP were studied using scanning electron microscope (SEM) images. As shown in Fig. 4 and S15, these functional silica nanoparticles (**SiNPs-DPA@Zn**^**2**+^ and **SiNPs-N**^+^) induce **SQ** to form fusiform and spherical assemblies (Fig. 4B and S15B). The average length of fusiform assemblies was 1.1 μm and the average diameter of spherical assemblies was 700 nm. As ATP was added to these assemblies, the supramolecular assemblies were converted to nano-assemblies (Fig. 4C and S15C). The sizes of self-assemblies were about 70 nm, which is a little larger than that of nanoparticles without **SQ** and ATP. The result suggests that the aggregates of **SQ** disappeared and ATP together with **SQ** was absorbed on the surface of nanoparticles during this process, and the thickness being composed of ATP and **SQ** on the outside of nanoparticles is about 5 nm.

### The investigation of selectivity of SQ-SiNPs-N^+^ and SQ-SiNPs-DPA@Zn^2+^ over various ions

**Figure 5:**
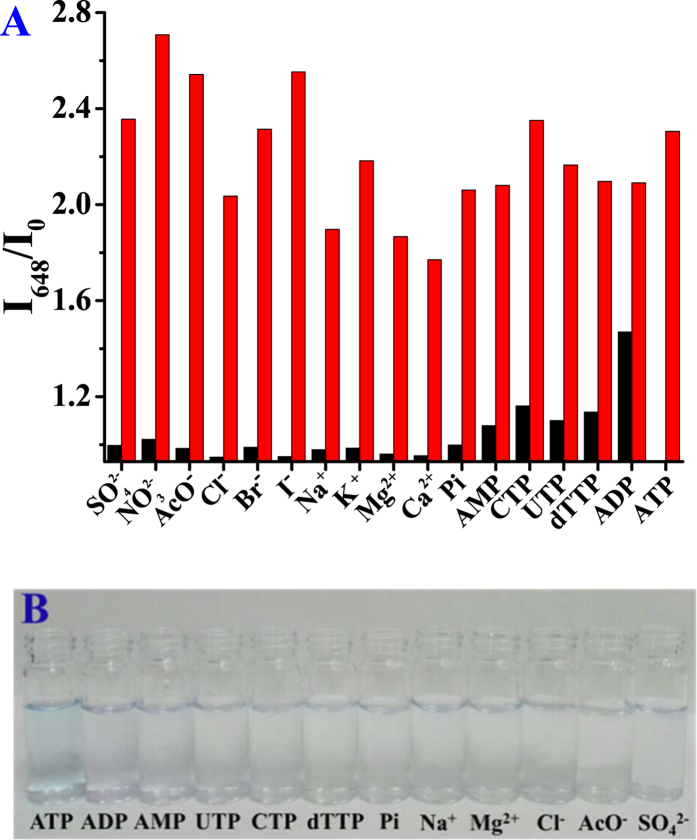
(**A**) The relative fluorescence intensity change of **SQ** (5.0 μM) at 648 nm in the presence of **SiNPs-DPA@Zn**^**2**+^ (220 mg/L) in PBS (10 mM, pH 7.2) upon addition 333 μM of various ions or other nucleoside polyphosphates (black bars). Red bars represent the relative intensity change with subsequent addition of ATP (333 μM). (**B**) Photographs of color change of **SQ** (5 mM) in PBS in the presence of **SiNPs-DPA@Zn**^**2**+^ (220 mg/L) upon addition of different ions (333 μM).

To evaluate the selectivity of these supramolecular assemblies, the fluorescence responses of **SQ-SiNPs-N**^+^ and **SQ**-**SiNPs-DPA@Zn**^**2**+^ solution to other phosphate anions (ADP, AMP, UTP, CTP and dTTP), common anions (Cl^−^, Br^−^, I^−^, SO_4_^2−^, NO_2_^2−^, AcO^−^ and Pi) and cations (Na^+^, K^+^, Ca^2+^ and Mg^2+^) were investigated. For **SQ-SiNPs-N**^+^ in water, although common ions did not induce any obvious fluorescence changes, nucleotide triphosphates (ATP, CTP, UTP and dTTP) caused obvious fluorescence responses (Fig. S16). For **SQ**-**SiNPs-DPA@Zn**^**2**+^ in buffer solution, except that ADP induced a slight fluorescence change there are no any obvious fluorescence changes upon addition all these ions (Fig. 5A). Moreover, only ATP triggers a color change observed by naked eyes (Fig. 5B). Due to the stronger binding of DPA-Zn^2+^ to ATP, the selectivity of supramolecular assemblies **SQ**-**SiNPs-DPA@Zn**^**2**+^ for ATP is better than that of **SQ-SiNPs-N**^+^. The more negatively charged phosphate anion, the stronger the intermolecular interaction that would occur, which is likely to be the reason of good selectivity for ATP over ADP and AMP. Furthermore, the selective π-π interaction between adenosine segment of ATP and **SQ** is accounted for the selective recognition of ATP over other nucleoside triphosphates (CTP, UTP and dTTP)^32^.

### The supramolecular assemblies of SQ-SiNPs-DPA@Zn^2+^ for ATP imaging in living cells

**Figure 6:**
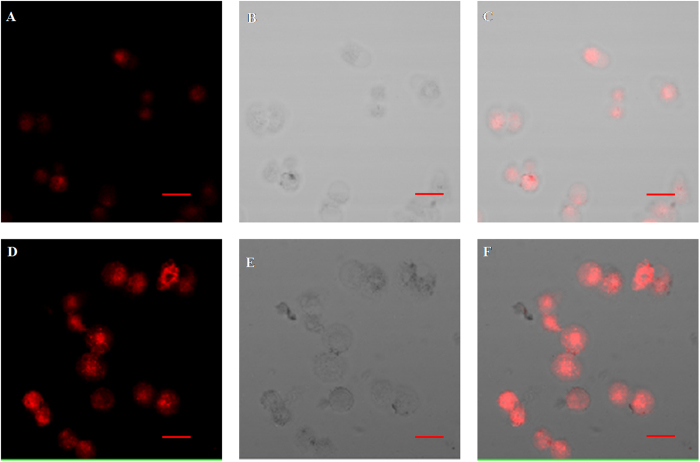
Confocal fluorescence images of MCF-7 cells. (**A**–**C**) images of MCF-7 cells pretreated with **SQ** (5 μM) for 10 min and subsequent treatment with **SiNPs-DPA@Zn**^**2**+^ (220 μg/ml) for 30 min; (**D**–**F**) images of MCF-7 cells pretreated with **SQ** (5 μM) for 10 min and subsequent treatment of **SiNPs-DPA@Zn**^**2**+^ (220 μg/ml) for 30 min, followed by treatment with **ATP** (50 μM) for 30 min; (A and D) images were taken in fluorescence field; (B and E) bright-field images of MCF-7 cells in samples; and (C and F) is the overlap of bright-field and fluorescence. Images were acquired by using excitation windows of λ_ex_ = 559 nm. Scare bar: 50 μm.

The supramolecular assemblies of **SQ-SiNPs-DPA@Zn**^**2**+^ were further performed to detect ATP in living cells. Fluorescence images were acquired *via* confocal microscopy. In this study, MCF-7 cells were used to incubate with **SQ-SiNPs-DPA@Zn**^**2**+^. As shown in Fig. 6, relative weak fluorescence signal occurred in the cells. However, obvious fluorescence enhancement was observed as the cells were further treated with ATP at 37 °C for 30 min. These cell experiments showed that **SQ-SiNPs-DPA@Zn**^**2**+^ could respond ATP in living cells.

## Methods

### Reagents and apparatus

Unless otherwise stated, all chemicals and reagents were obtained from commercial suppliers and used without further purification. 3-Chloropropyltriethoxysilane, sodium iodide, triton X-100, tetraethylorthosilicate (TEOS), N-trimethoxysilylpropyl-N,N,N-trimethylammonium chloride and zinc chloride were purchased from Aladdin Industrial Inc (Shanghai, China). Cyclohexane, *n*-hexano, ammonium hydroxide (NH_3_·H_2_O, 25% w/w in water), ethanol, methanol, acetonitrile, acetone and dichloromethane were purchased from KeLong (Chengdu, China). Methanol and acetonitrile were purified and redistilled by standard methods prior to use. Water used was deionized.

Cationic squaraine dye (**SQ**)^33^ and di-(2-picolyl)amine (DPA)^34^,^35^ were synthesized and purified as reported previously.

^1^H NMR and ^13^C NMR were collected on a Bruker 500 avance III spectrometer. Mass spectrometric (MS) data were obtained with HP1100LC/MSD MS instruments. Absorption and emission spectra were collected by using a Shimadzu 1750 UV-visible spectrometer and a RF-5301 fluorescence spectrometer (Japan), respectively. SEM and TEM images were observed with a JSM-6701F scanning electron microscope and HT7700 transmission electron microscope, respectively. Diameter distribution was obtained with Delsa Nano C analyzer (Beckman Coulter, Inc). Zeta potential measurements were performed with ZetaPALS 51276 (Brookhaven Instruments Corp. New York). X-ray photoelectron spectroscopy (XPS) measurements were performed with an Escalab 250Xi (Thermol Scientific, US) spectrometer using Al k_α_ excitation radiation (1486.6 eV).

**SiNPs-DPA@Zn**^**2**+^ (20 mg/L) and **SiNPs-N**^+^ (6 mg/L) were dispersed in water as stock solution. Stock solution of **SQ** (5.0 × 10^−4^ M) was prepared in EtOH and further diluted to 5.0 × 10^−6^ M for titration experiments. Stock solutions of ATP, ADP, AMP, GTP, CTP, UTP, dTTP, Pi and metal ions were prepared in deionized water, and the concentrations are fixed to 10 × 10^−2^ M.

### Cell culture and fluorescence image

MCF-7 cells were seeded on 35 mm glass-bottomed dishes (NEST) and incubated in RPMI-1640 in an incubator (37 °C, 5% CO_2_ and 20% O_2_) for 24 h. The cells were rinsed slightly 3 times with fresh RPMI-1640 and incubated in RPMI-1640 medium spiked with SQ (5 μM) for 10 min. After washing with fresh RPMI-1640, the cells were incubated in fresh RPMI-1640 containing of **SiNPs-DPA@Zn**^**2**+^ (220 μg/ml) for 30 min. After washing with fresh RPMI-1640, the cells were further incubated in fresh RPMI-1640 containing of **ATP** (50 μM) for 30 min. Cells were then analyzed by Laser Scanning^36^ Confocal Microscope (AIR).

### Synthesis of DPA-Si

The mixture of di-(2-picolyl)amine (1.141 g, 5.72 mmol), 3-chloropropyltriethoxysilane (1.38 g, 5.72 mmol), NaI (2.13 g, 11.44 mmol) and K_2_CO_3_ (1.58 g, 11.44 mmol) in acetonitrile was heated to reflux and stirred for 24 h. The mixture was allowed to cool to room temperature and evaporated to dryness under reduced pressure. The crude product was purified through flash column chromatography (CH_2_Cl_2_) to obtain faint yellow oil product, yield 76%. ^1^H NMR (500 MHz, CDCl_3_) δ 8.45 (d, *J* = 4.8 Hz, 2 H), 7.58 (td, *J* = 7.7 Hz, 2 H), 7.51 (t, *J* = 6.2 Hz, 2 H), 7.08 (m, 2 H), 3.78 (d, *J* = 4.9 Hz, 4 H), 3.72 (m, 5 H), 3.65 (m, 1 H), 2.56–2.48 (m, 2 H), 1.66–1.57 (m, 2 H), 1.18–1.11 (m, 9 H), 0.53 (m, 2 H). *m/z* (TOF-LD): Calcd. [M + H]^+^ For C_21_H_34_N_3_O_3_Si: 404.59, found: 404.24.

### Preparation of SiNPs-DPA@Zn^2+^

The W/O microemulsion was prepared firstly at ambient temperature by mixing 1.77 mL of Triton X-100, 7.5 mL of cyclohexane, 1.8 mL of *n*-hexanol and 500 μL of water for 24 h^37^. In the presence of 100 μL of TEOS, a polymerization reaction was initiated by adding 60 μL of NH_3_·H_2_O. The reaction was allowed to continue for 12 h. Then the other 25 μL of TEOS, 38 μL of **DPA-Si** compound, and 50 μL of NH_3_·H_2_O were added into the system, the mixture was stirred for other 12 h. After the reaction was completed, the nanoparticles were isolated by acetone, followed by centrifuging and washing with ethanol and water several times to remove any surfactant molecules. These as-prepared nanoparticles were refluxed with excess ZnCl_2_ in H_2_O for 3 h. After the reaction was completed, the nanoparticles were centrifuged and washed three times with water to remove excess ZnCl_2_. The **SiNPs-DPA@Zn**^**2**+^ were redispersed in H_2_O and stored in 4 °C for use.

### Preparation of SiNPs-N^+^

The reversed-phase microemulsion was prepared as above mentioned. In the presence of 100 μL of TEOS, a polymerization reaction was initiated by adding 60 μL of NH_3_·H_2_O. The reaction was allowed to continue for 12 h. Then the other 25 μL of TEOS and 30 μL of N-Trimethoxysilylpropyl-N,N,N-trimethylammonium chloride, 50 μL of NH_3_·H_2_O were added into the system, the mixture was stirred for other 12 h. After the reaction was completed, the nanoparticles were isolated by acetone, followed by centrifuging and washing with ethanol and water several times to remove any surfactant molecules. The **SiNPs-N**^+^ were redispersed in H_2_O and stored in 4 °C for use.

## Summary

In summary, three kinds of silica nanoparticles with or without functional groups were synthesized. Either SiNPs or positively charged silica nanoparticles have the ability of inducing positively charged squaraine to aggregate in aqueous solution to form supramolecular assemblies. Different from the reported systems dependent on electrostatic interactions, these assemblies are presumed to rely on hydrophobic and ion-polar interactions. Silica nanoparticles direct **SQ** to aggregate along their surface to form fusiform and spherical assemblies, which is proved by SEM images. Furthermore, the supramolecular assemblies showed dynamic shift under treatment with small molecule ATP. ATP as building blocks was absorbed on the surface of silica nanoparticles through metal-anion coordination and electrostatic interactions, in which the aggregates of **SQ** was transferred to monomer. The thickness being composed of ATP and **SQ** on the outside of nanoparticles is about 5 nm. Through the selective π-π interaction between adenosine segment of ATP and **SQ**, these supramolecular assemblies showed selective turn-on fluorescence response to ATP in NIR region over other ions. Due to the stronger binding of DPA-Zn^2+^, the selectivity of supramolecular assemblies **SQ**-**SiNPs-DPA@Zn**^**2**+^ for ATP is better than that of **SQ-SiNPs-N**^+^. The creation of supramolecular assembiles induced by silica nanoparticles provides a proof-of-principle method for applications in the field of construction of optical devices, molecular sensor design and simulation of functional biomolecules.

## Additional Information

**How to cite this article**: Feng, R. *et al*. Hierarchical self-assembly of squaraine and silica nanoparticle functionalized with cationic coordination sites for near infrared detection of ATP. *Sci. Rep.* **7**, 43491; doi: 10.1038/srep43491 (2017).

**Publisher's note:** Springer Nature remains neutral with regard to jurisdictional claims in published maps and institutional affiliations.

## Supplementary Material

Supplementary Information
