# Supplementary material for: Hierarchical self-assembly of squaraine and silica nanoparticle functionalized with cationic coordination sites for near infrared detection of ATP
Source: Sci Rep. 2017 Feb 27;7:43491. doi: 10.1038/srep43491 (PMC5327475; doi:10.1038/srep43491)
Supplement: Supplementary Information [file srep43491-s1.pdf]

Hierarchical self-assembly of squaraine and silica nanoparticle functionalized with cationic coordination sites for near infrared detection of ATP

Ruizhi Feng, Weining Shi, Dejia Wang, Jia Wen, Hongjuan Li, Shiguo Sun and Yongqian Xu*

Shaanxi Key Laboratory of Natural Products & Chemical Biology, College of Chemistry & Pharmacy, Northwest A&F University, Yangling, Shaanxi, 712100, P. R. China.

**Corresponding author:*

Yongqian Xu (xuyq@nwsuaf.edu.cn)

Synthetic route of DPA-Si

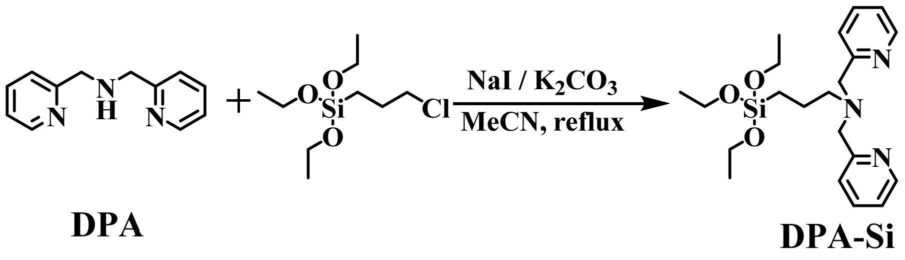

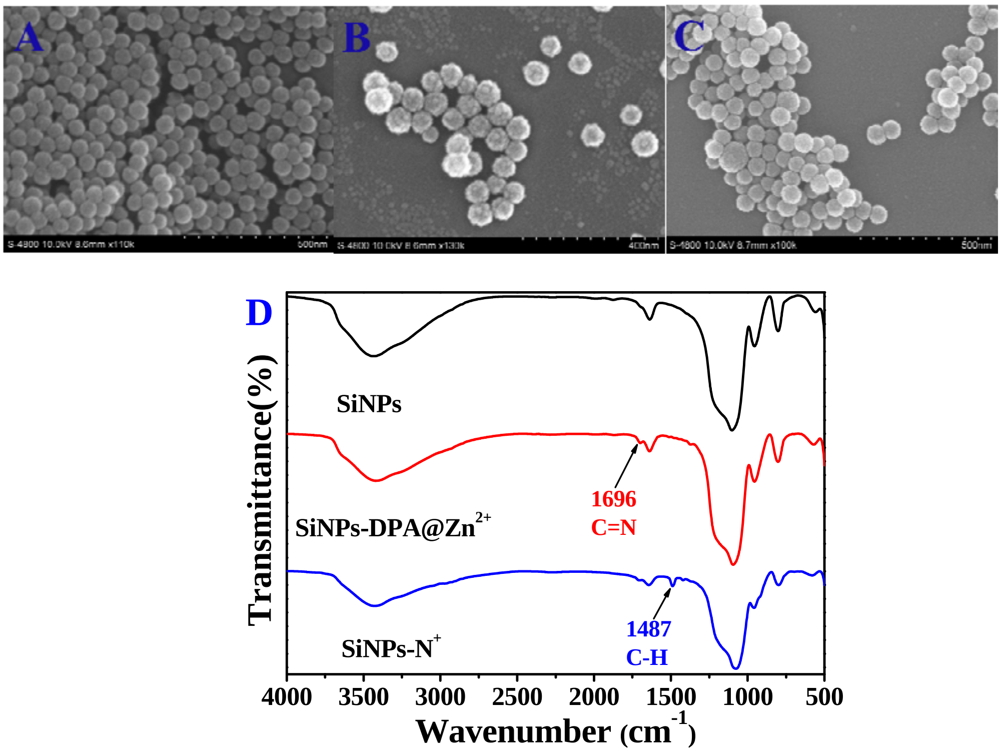

Fig. S1 SEM spectra of SiNPs (A), **SiNPs-DPA@Zn^{2+}** (B) and **SiNPs-N^{+}** (C), and (D) FTIR spectra of SiNPs, **SiNPs-DPA@Zn^{2+}** and **SiNPs-N^{+}**.

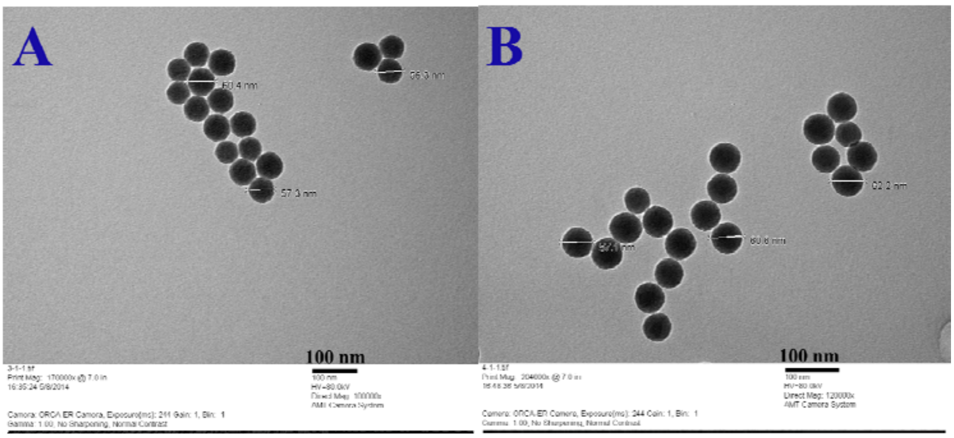

Fig. S2 TEM spectra of **SiNPs-DPA@Zn^{2+}** (A) and **SiNPs-N^{+}** (B).

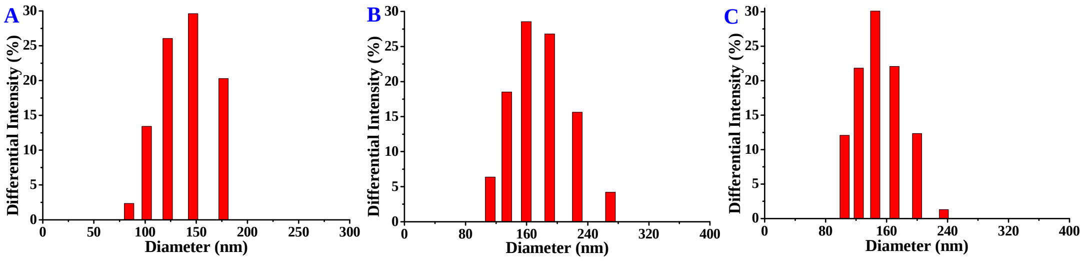

Fig. S3 Dynamic light scattering (DLS) spectra of **SiNPs** (A), **SiNPs-DPA@Zn^{2+}** (B) and **SiNPs-N^{+}** (C).

Samples	(A) **SiNPs**	(B) **SiNPs-DPA@Zn^{2+}**	(C) **SiNPs-N^{+}**
Zeta potential (mV)	-32.39	18.63	15.65

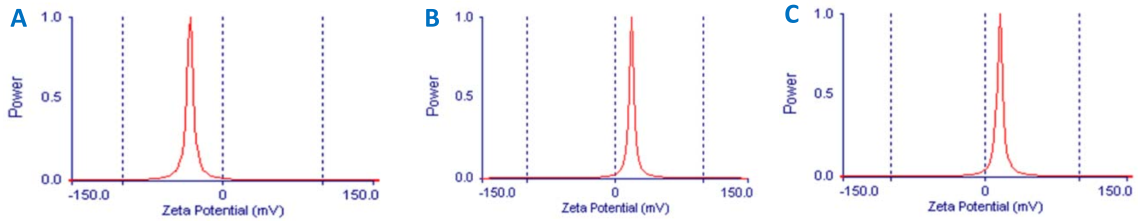

Fig. S4 Zeta potential analysis of **SiNPs** (A), **SiNPs-DPA@Zn^{2+}** (B) and **SiNPs-N^{+}** (C).

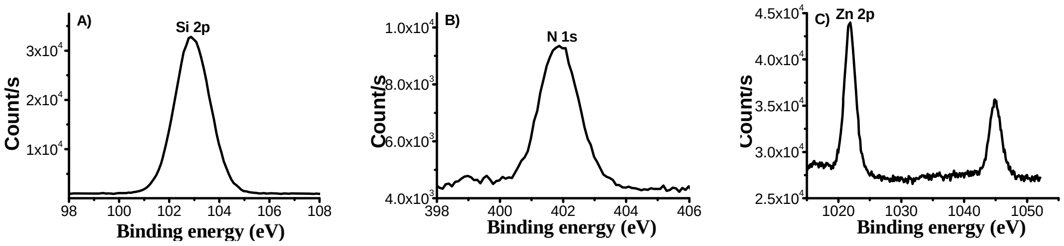

Fig. S5 XPS spectra of **SiNPs** (A), **SiNPs-DPA@Zn^{2+}** (B) and **SiNPs-N^{+}** (C).

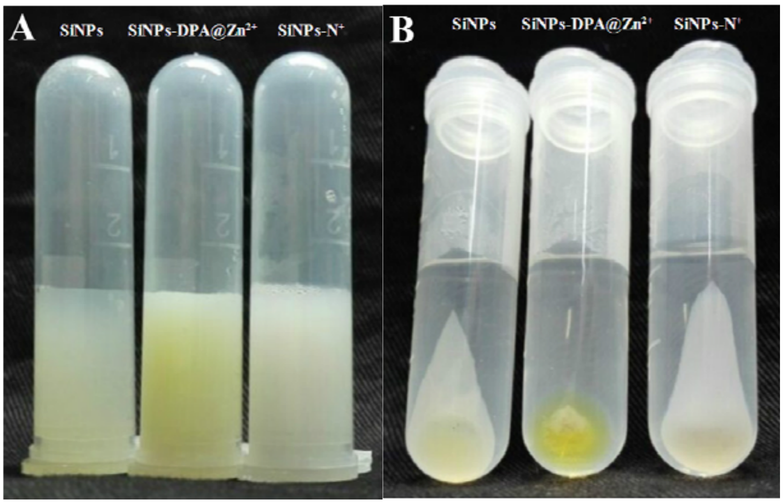

Fig. S6 The photographs of **SiNPs**, **SiNPs-DPA@Zn^{2+}** and **SiNPs-N^{+}** before (A) and after (B) centrifugation.

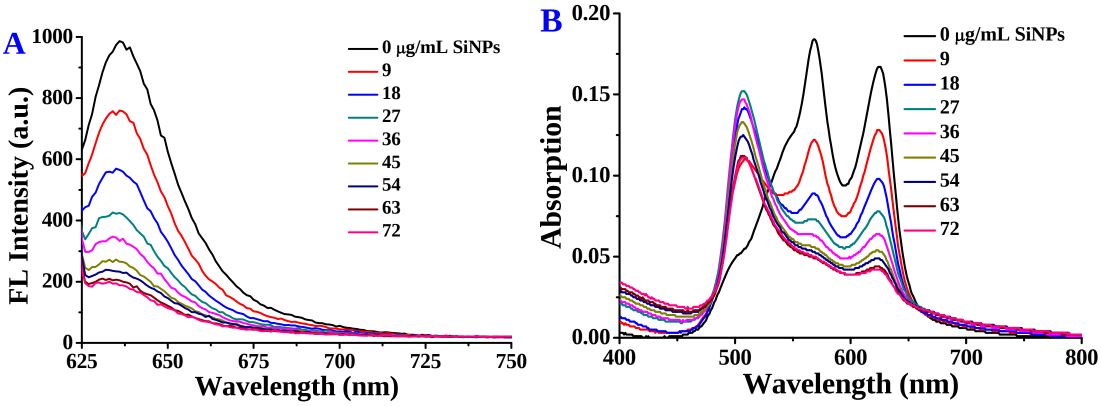

Fig. S7 Fluorescence spectra and UV-Vis absorption spectra changes of **SQ** (5 μM) in PBS (10 mM, pH 7.2) upon addition of **SiNPs**.

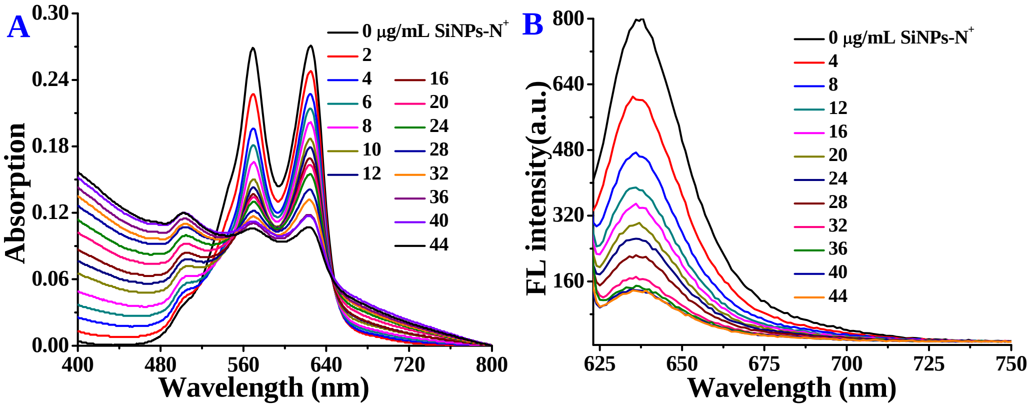

Fig. S8 UV-Vis absorption spectra (A) and fluorescence spectra (B) changes of **SQ** (5 μM) in water upon addition of **SiNPs-N^{+}**.

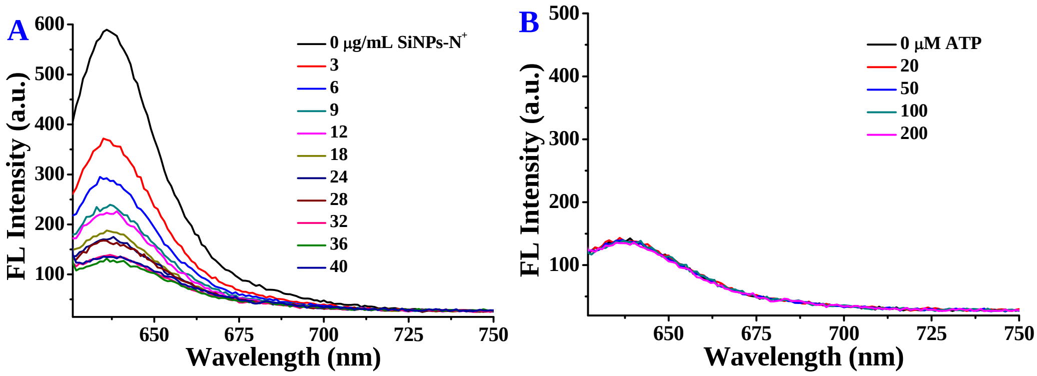

Fig. S9 Fluorescence spectra change of **SQ** (5 μM) in PBS (10 mM, pH 7.2) upon addition of **SiNPs-N$^+$** (A) and sequential addition of ATP (B).

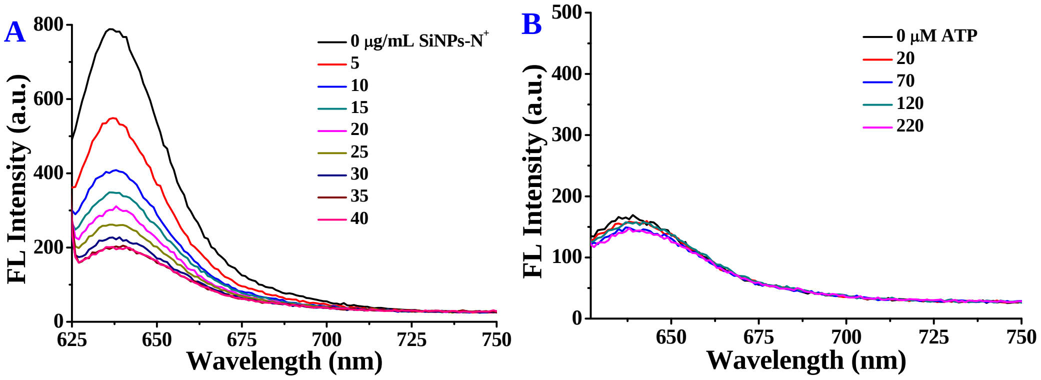

Fig. S10 Fluorescence spectra change of **SQ** (5 μM) in HEPES buffer solution (10 mM, pH 7.2) upon addition of **SiNPs-N$^+$** (A) and sequential addition of ATP (B).

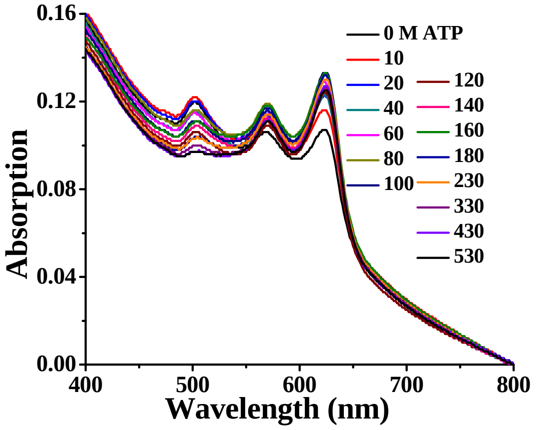

Fig. S11 UV-Vis absorption spectral changes of **SQ** (5 μM) in the presence of **SiNPs-N$^+$** (44 mg/L) in water upon addition of ATP.

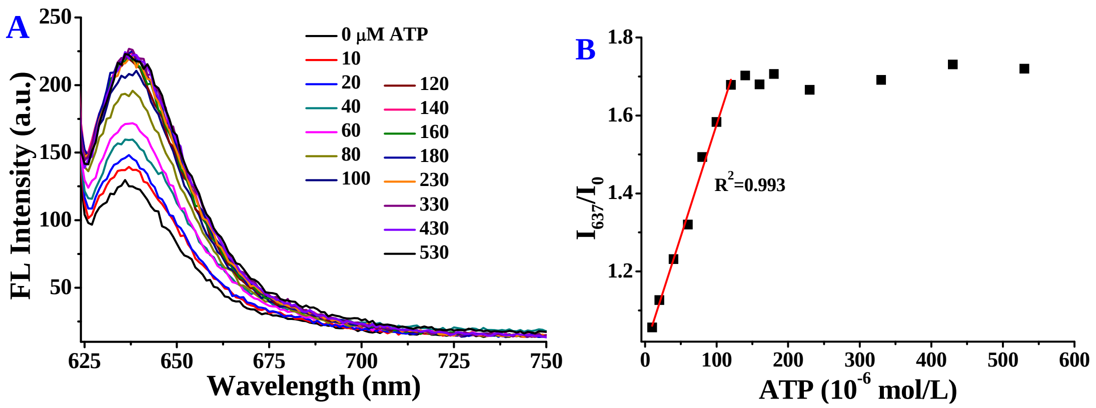

Fig. S12 Fluorescence spectra change (A) and relative fluorescence intensity change (I_{637}/I_0) (B) of **SQ** (5 μM) in the presence of **SiNPs-N$^+$** (44 mg/L) in water upon addition of ATP.

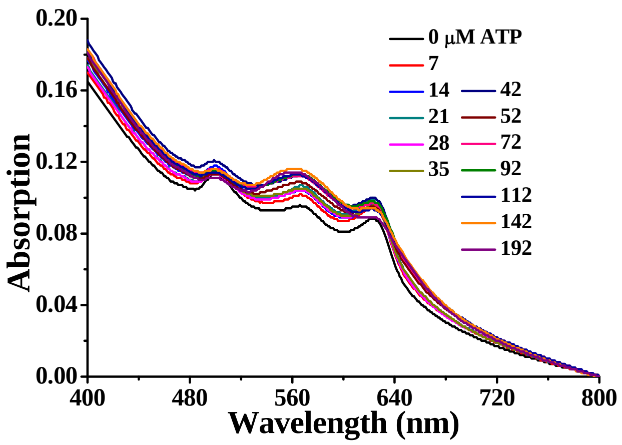

Fig. S13 UV-Vis absorption spectra change of **SQ** (5 μM) in the presence of **SiNPs-DPA@Zn^{2+}** (220 mg/L) in PBS (10 mM, pH=7.2) upon addition of ATP.

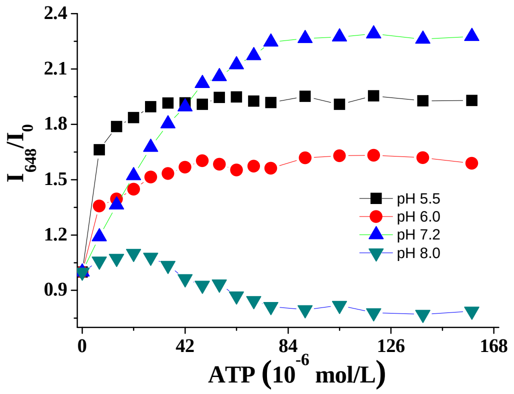

Fig. S14 Relative fluorescence intensity change (I_{648}/I_0) of **SQ** (5 μM) in the presence of **SiNPs-DPA@Zn^{2+}** (220 mg/mL) in PBS (10 mM) with different pH values (5.5, 6.0, 7.2, 8.0) upon addition of ATP.

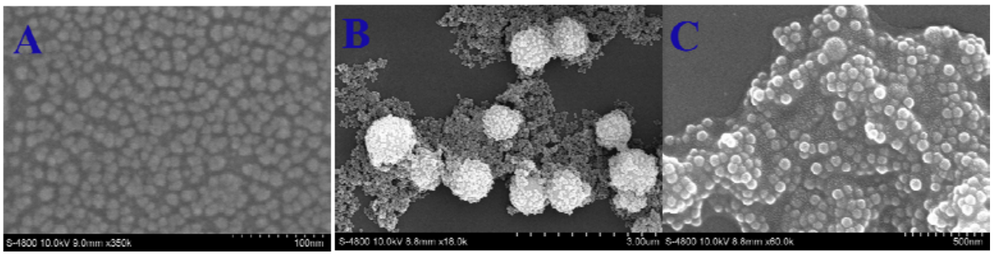

Fig. S15 SEM images of **SQ** (A) in the presence of **SiNPs-N$^+$** before (B) and after (C) addition of ATP.

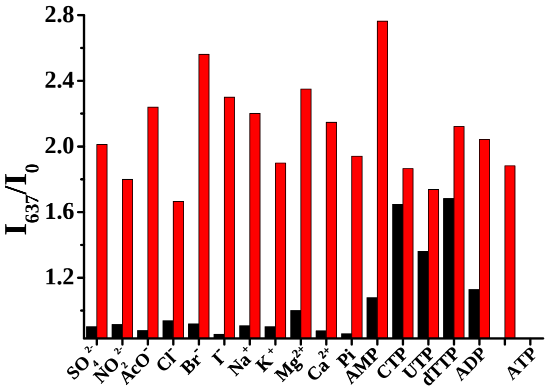

Fig. S16 The relative fluorescence intensity change of **SQ** (5.0 μM) at 637 nm in the presence of **SiNPs-N$^+$** (44 μg/mL) in water upon addition of various ions or other nucleoside polyphosphates (333 μM) (black bars). Red bars represent the relative intensity change with subsequent addition of ATP (333 μM).

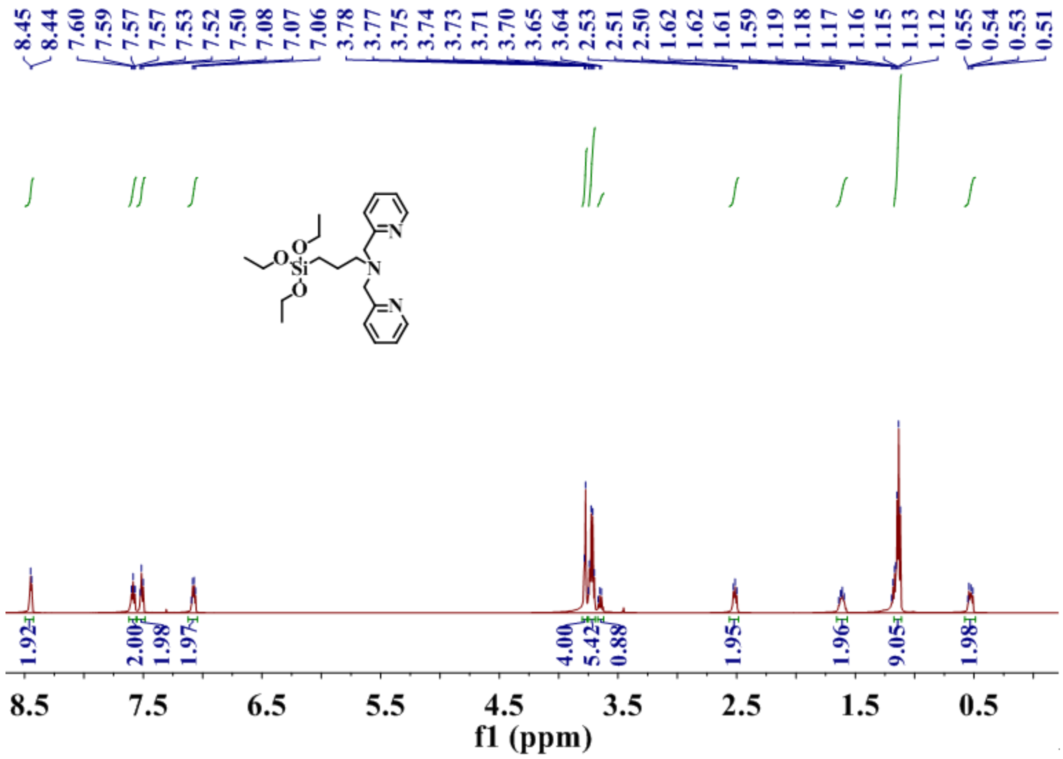

Fig. S17 ^{1}H-NMR Spectrum of compound **DPA-Si** in $CDCl_3$

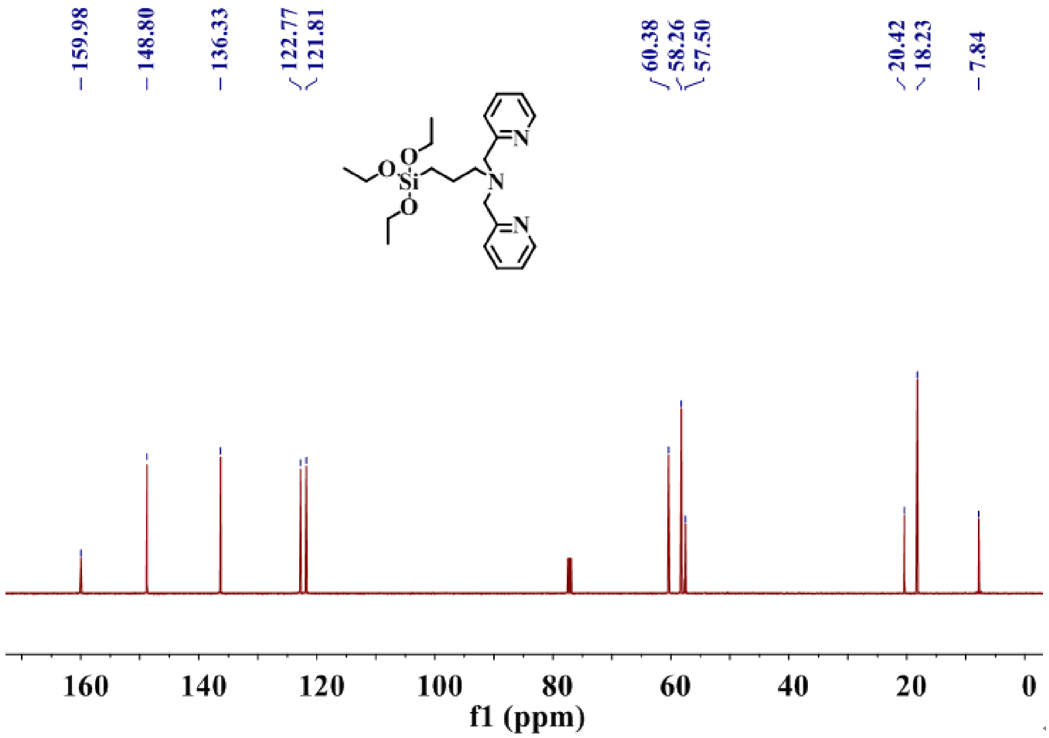

Fig. S18 ^{13}C-NMR Spectrum of compound **DPA-Si** in $CDCl_3$.

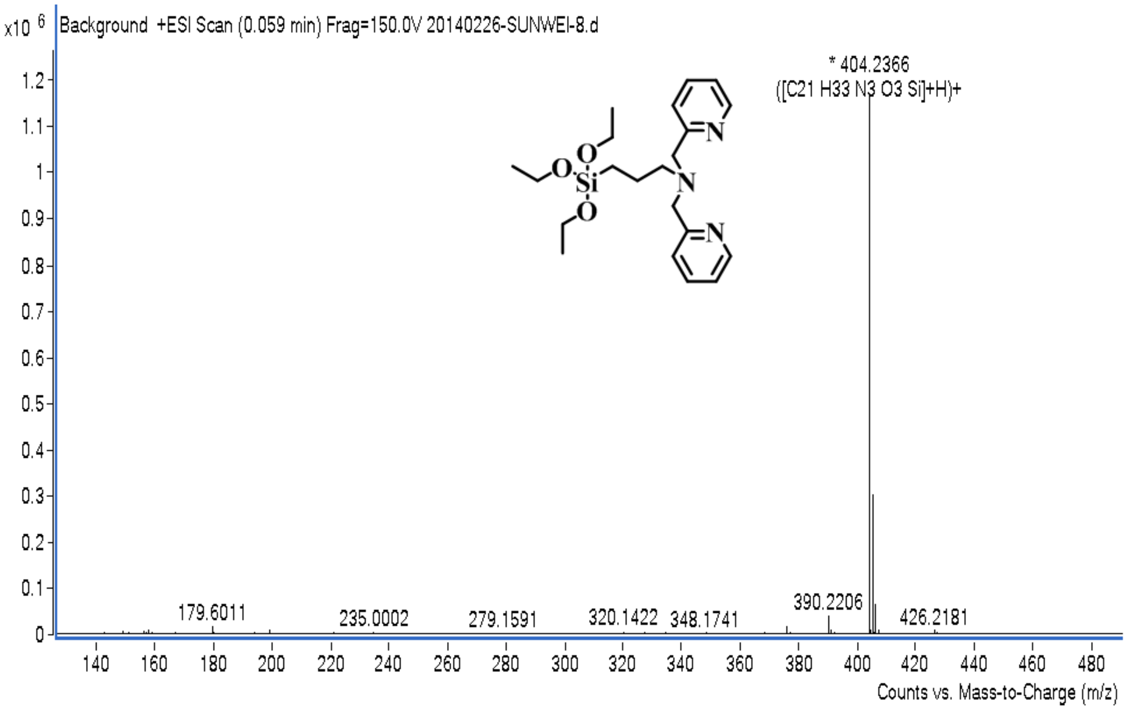

Fig. S19 MS of compound **DPA-Si**.
